# Effects of angiotensin-converting enzyme inhibitors and angiotensin receptor blockers on cardiovascular events and residual renal function in dialysis patients: a meta-analysis of randomised controlled trials

**DOI:** 10.1186/s12882-017-0605-7

**Published:** 2017-06-30

**Authors:** Youxia Liu, Xinxin Ma, Jie Zheng, Junya Jia, Tiekun Yan

**Affiliations:** 1 Department of Nephrology, General Hospital of Tianjin Medical University, NO. 154, Anshan road, Heping District, Tianjin, China; 2 Division of Nephrology, Department of Medicine, The Third Affiliated Hospital of Sun Yat-sen University, Guangzhou, People’s Republic of China; 3 Radiology Department, General Hospital of Tianjin Medical University, Tianjin, People’s Republic of China

**Keywords:** Angiotensin-converting enzyme inhibitors, Angiotensin receptor blockers, Cardiovascular events, Residual renal function, Dialysis, Meta-analysis

## Abstract

**Background:**

The role of angiotensin-converting enzyme inhibitors (ACEIs) and angiotensin receptor blockers (ARBs) reducing risk of cardiovascular events (CVEs) and preserving kidney function in patients with chronic kidney disease is well-documented. However, the efficacy and safety of these agents in dialysis patients is still a controversial issue.

**Methods:**

We systematically searched MEDLINE, Embase, Cochrane Library and Wanfang for randomized trials. The relative risk (RR) reductions were calculated with a random-effects model. Major cardiovascular events, changes in GFR and drug-related adverse events were analyzed.

**Results:**

Eleven trials included 1856 participants who were receiving dialysis therapy. Compared with placebo or other active agents groups, ARB therapy reduced the risk of heart failure events by 33% (RR 0.67, 95% CI 0.47 to 0.93) with similar decrement in blood pressure in dialysis patients. Indirect comparison suggested that fewer cardiovascular events happened during treatment with ARB (0.77, 0.63 to 0.94). The results indicated no significant differences between the two treatment regimens with regard to frequency of myocardial infarction (1.0, 0.45 to 2.22), stroke (1.16, 0.69 to 1.96), cardiovascular death (0.89, 0.64 to 1.26) and all-cause mortality (0.94, 0.75 to 1.17). Five studies reported the renoprotective effect and revealed that ACEI/ARB therapy significantly slowed the rate of decline in both residual renal function (MD 0.93 mL/min/1.73 m^2^, 0.38 to 1.47 mL/min/1.73 m^2^) and urine volume (MD 167 ml, 95% CI 21 ml to 357 ml). No difference in drug-related adverse events was observed in both treatment groups.

**Conclusions:**

This study demonstrates that ACE-Is/ARBs therapy decreases the loss of residual renal function, mainly for patients with peritoneal dialysis. Overall, ACE-Is and ARBs do not reduce cardiovascular events in dialysis patients, however, treatment with ARB seems to reduce cardiovascular events including heart failure. ACE-Is and ARBs do not induce an extra risk of side effects.

**Electronic supplementary material:**

The online version of this article (doi:10.1186/s12882-017-0605-7) contains supplementary material, which is available to authorized users.

## Background

Cardiovascular events (CVEs) are the leading causes of death among dialysis patients, with mortality rates 7 to 30 times higher than in the general population [1, 2]. Observational studies to date in dialysis patients have reported an association between progressive loss of residual glomerular filtration rate (GFR) and increased mortality [3, 4]; Causality has not been established with dialysis patient survival and residual renal function (RRF). Treatment with angiotensin-converting enzyme inhibitors (ACEIs) and angiotensin receptor blockers (ARBs) has provided significant cardiovascular protection and preserved RRF for chronic kidney disease (CKD) patients [5–7]. Unfortunately, most trials excluded patients with end stage renal disease (ESRD) receiving maintenance dialysis, the beneficial effects of ACEI/ARBs on CVEs and RRF in dialysis patients remain uncertain. Some large-scale trials tested the effects of ACEIs/ARBs therapy in dialysis patients provided inconsistent results, and much uncertainty persists regarding the protective effects of this agent [8–11].

We therefore undertook a meta-analysis to evaluate the effect of ACEIs and ARBs on cardiovascular events and residual renal function decline in patients receiving dialysis.

## Methods

### Date sources, search strategy and selection criteria

We undertook a systematic review of the literature according to the approach recommended by the statement for the conducting of meta-analysis of intervention studies [12]. Relevant studies were identified by searching the following data sources: MEDLINE (OVID) (from 1950 to December 2016), Embase (from 1970 to December 2016), the Cochrane Library database (Cochrane Central Register of Active controlled Trials; no date restriction), and Wanfang database. We used the MeSH headings and text words of all spellings of known ACE inhibitors and ARBs, dialysis, cardiovascular events, and kidney failure (see Additional files 1). Trials were limited to randomized controlled trials (RCTs) without language restriction. Reference lists from identified trials and review articles were searched manually to identify any other relevant studies. We also searched the Clinical Trials.gov website for randomized trials that were registered as completed but not yet published. All completed RCTs that assessed the effects of ACE-Is or ARBs compared with placebo or other antihypertensive drugs in dialysis patients, and which reported cardiovascular, renal or adverse outcomes, were eligible for inclusion.

### Data extraction and quality assessment

Published reports were obtained for each eligible trial, and relevant information extracted into a spreadsheet. The data sought included dialysis modality, number of patients, country in which the study was performed, patient age, mean baseline systolic and diastolic blood pressure values, residual GFR, Kt/v, mean duration on dialysis, follow-up duration, change in blood pressure, outcome events (including CVEs, all-cause death, and RRF). Major cardiovascular events were defined as a composite of fatal or non-fatal myocardial infarction, fatal or non-fatal stroke, heart failure, or comparable definitions used by individual authors or cardiovascular mortality. Residual renal function was measured by GFR or endogenous creatinine clearance (CrCl), or urine volume, and drug-related adverse events if sufficient data were available. The literature were searched and identified by two investigators (LYX and MXX) independently. Data extraction and quality assessment (Grading of Recommendations Assessment, Development and Evaluation system) [13] was undertaken independently by two investigators (ZJ and MXX) using a standardized approach. Any disagreement between the two investigators in the abstracted data was adjudicated by a third reviewer (JJY).

### Statistical analysis

The relative risk (RR) and 95% confidence interval (CI) for each outcome was calculated before pooling by the random-effects model. For the continuous measurement of change of GFR, blood pressure and urine volume, we used the weighted mean difference between groups. Heterogeneity across the included studies was analyzed using the I^2^ to describe the percentage of variability. We made graphic representations of potential publication bias using Begg Funnel plots of the natural logarithm of the RR versus its standard error (SE) and assessed them visually. A 2-sided *p* value less than 0.05 was considered statistically significant, and statistical analyses were performed using STATA, version 12.0 and Review Manager 5.1.

## Results

**Fig. 1:**
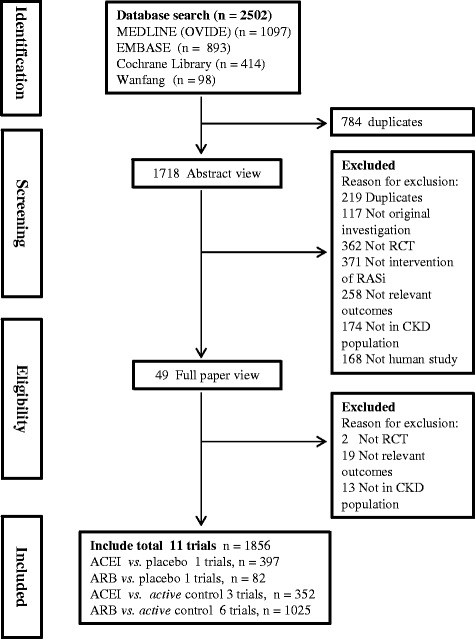
Process for identifying studies eligible for the meta-analysis

Our literature search yielded 2502 relevant articles, of which 49 were reviewed in full text (Fig. 1). A total of 11 relevant RCTs with 1856 patients were included for further analysis [8–11, 14–20]. The characteristics of the included studies are presented in Table 1. One trial (*n* = 397) compared ACE-Is with placebo [9], one compared ARBs with placebo (*n* = 82) [11], three studies (*n* = 352) compared ACE-Is with active control [14, 18, 20], and 6 studies (*n* = 1025) compared ARBs with active control [8, 10, 15–17, 19]. These studies were performed between 2003 to 2014 with sample sizes ranging from 32 to 469, and the mean follow-up was 3.8 years. Seven trials with 1686 patients undergoing hemodialysis and four trials including 170 patients with peritoneal dialysis were included.

**Table 1:** Characteristics of studies in meta-analysis

Trials	Treatment	Dialysis modality	Country	No. patients	Age,years	Mean Baseline SBP,mmHg	Mean Baseline DBP,mmHg	Residual GFR,mL/min per 1.73 m2	Kt/V	Mean Duration on dialysis,months	follow-up, years
ACE-Is vs. placebo											
FOSIDIAL 2006 [9]	ACE-I/Placebo	HD	France	397	67	146	77		1.3	49.2	2
ARBs vs. placebo											
SAFIR 2014 [11]	ARB/Placebo	HD	Denmark	82	61	146	76	5.2		4.6	1
ACE-Is vs. active control											
Yilmaz 2010 [18]	ACE-I/CCB	HD	Turkey	92	53.8	157	88		1.4	47	1
Philip 2003 [14]	ACE-I/Conventional ahtihypertensive agents	PD	China Kong Hong	60	58	151	83.5	3.55	2.08	10.5	1
HDPAL 2014 [20]	ACE-I/atenolol	HD	USA	200	53.1	151	87.1				1
ARBs vs. active control											
Suzuki 2008 [10]	ARB/Conventional ahtihypertensive agents	HD	Japan	366	59.5	155	81		1.1	44.4	3
Takahashi 2006 [8]	ARB/CCB	HD	Japan	80	61	153	82			33.1	1.6
OCTOPUS 2013 [19]	ARB/Conventional ahtihypertensive agents	HD	Japan	469	59	159	80		1.2	88	3.5
Suzuki 2004 [10]	ARB/CCB	PD	Japan	34	63.5	165	76	4.3	1.97		24
Wang J	ARB/Conventional ahtihypertensive agents	PD	China	32	42	158	102	4.8	2.09	29	2.4
Zhong H	ARB/CCB	PD	China	44	45	134	83	4.5	1.97		1

Abbreviations: ACEI, angiotensin-converting enzyme inhibitors; ARB, angiotensin receptor blockers; CCB, calcium channel blockers; GFR, glomerular filtration rate; HD, hemodialysis; PD, peritoneal dialysis

The quality of the included studies was estimated using the Cochrane Collaboration tool for assessing the risk of bias; low versus high risk of bias is indicated for each study in Table 2.

**Table 2:** Quality assessment for included trials

Trial	Sequence generation	Allocation concealment	Blinding			Incomplete outcome data	Selective outcome reporting	Other source of bias
			participants	personnel	outcome assessors			
FOSIDIAL 2006 [9]	LOW	LOW	LOW	LOW	LOW	LOW	LOW	LOW
SAFIR 2014 [11]	LOW	UNCLEAR	LOW	LOW	UNCLEAR	LOW	LOW	LOW
Yilmaz 2010 [18]	UNCLEAR	UNCLEAR	HIGH	HIGH	HIGH	LOW	LOW	LOW
Philip 2003 [14]	LOW	LOW	HIGH	HIGH	HIGH	LOW	LOW	LOW
HDPAL 2014 [20]	LOW	LOW	HIGH	HIGH	HIGH	LOW	LOW	LOW
Suzuki 2008 [10]	LOW	LOW	HIGH	HIGH	HIGH	LOW	LOW	LOW
Takahashi 2006 [8]	LOW	LOW	HIGH	HIGH	LOW	LOW	UNCLEAR	UNCLEAR
OCTOPUS 2013 [19]	UNCLEAR	UNCLEAR	HIGH	HIGH	LOW	LOW	LOW	LOW
Suzuki 2004 [15]	LOW	LOW	HIGH	HIGH	HIGH	LOW	LOW	LOW
Wang J [16]	UNCLEAR	UNCLEAR	UNCLEAR	UNCLEAR	UNCLEAR	LOW	LOW	UNCLEAR
Zhong H [17]	UNCLEAR	UNCLEAR	UNCLEAR	UNCLEAR	UNCLEAR	LOW	LOW	UNCLEAR

Assessment of risk bias according to the Cochrane Collaboration’s tool, low risk of bias was represented as “LOW” and high risk of bias was “HIGH”

There was no significant difference in blood pressure over time between patients treated with ACEI/ARB and those treated with placebo or other antihypertensive drugs (MD −1.11 mmHg, 95% CI -2.55 to 0.32 mmHg; *p =* 0.13; and MD 0.83 mmHg, 95% CI -0.68 to 2.35 mmHg; *p =* 0.28; for systolic and diastolic blood pressure, respectively).

### Cardiovascular events

**Fig. 2:**
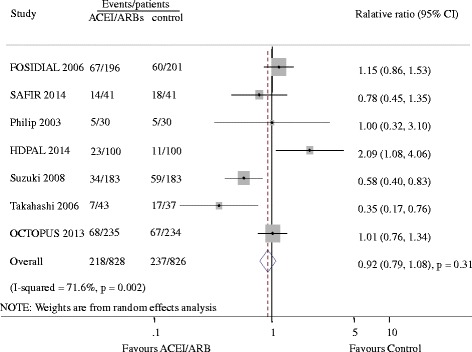
Effect of ACE-Is or ARBs compared with placebo or other active agents on cardiovascular events

**Fig. 3:**
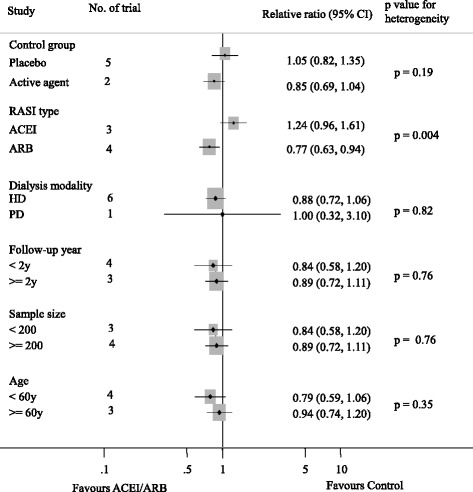
Subgroup analysis for the relationship between CVE and the use of ACEI/ARB

**Fig. 4:**
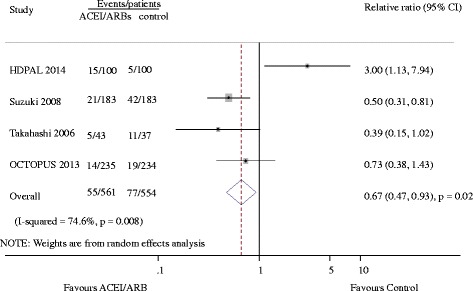
Effect of ACE-Is or ARBs compared with placebo or other active agents on heart failure

**Fig. 5:**
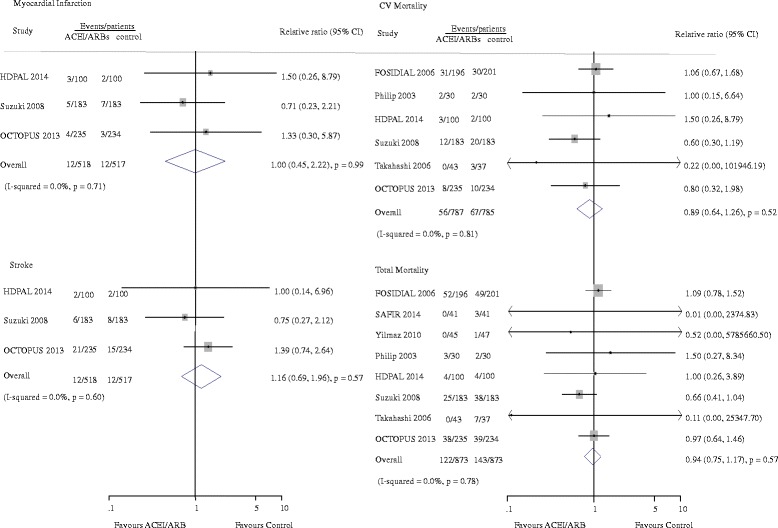
Effect of ACE-Is or ARBs compared with placebo or other active agents on myocardial infarction, stroke, CV motality and total mortality

Seven studies reported 455 cardiovascular events [8–11, 14, 19, 20]. Of the 828 patients treated with ACEI/ARB there were 218 cardiovascular events (26.3%) and 237 events occurred in 826 patients treated with placebo or active agents (28.7%). Overall, ACE-Is and ARBs did not reduce cardiovascular events versus placebo or other antihypertensive agents (RR 0.92, 95% CI 0.79 to 1.08, Fig. 2). There was evidence of significant heterogeneity for effect of CVEs across included studies (I^2^ = 71.6%, *p =* 0.002). Subgroup analysis indicated that the presence of heterogeneity was due to the different RASI category (ACEI or ARB), shown in Fig. 3. Indirect comparison suggested that ARB seemed to provided a higher probability of being beneficial for CVEs in dialysis patients (0.77, 0.63 to 0.94), while ACEI did not (1.24, 0.96 to 1.61). Subgroup analysis detected no significant difference between the two groups with regard to different control group (placebo or active agents), the dialysis mode, follow-up year, sample size and patient age. Data for heart failure events were available from 4 trials including 1115 patients in whom 132 events were recorded [8, 10, 19, 20]. ACEI/ARB therapy in dialysis patients reduced the risk of heart failure events by 33% (0.67, 0.47 to 0.93) with extensive heterogeneity in the results of individual trials (I^2^ = 74.6%, *p =* 0.008, Fig. 4). In order to diminish the heterogeneity, a subgroup analysis was performed based on different type of RASI for comparison which led to a nearly 70% decrease of I^2^ while did not affect the association of ACEI/ARB with lower risk of heart failure events (0.22, 0.38 to 0.78; *p =* 0.001; I^2^ = 0.0%, *p =* 0.51). There were no significant differences between ACEI/ARB and placebo or active agents therapy on the outcomes of myocardial infarction (1.0, 0.45 to 2.22; I^2^ = 0%, *p =* 0.71), stroke (1.16, 0.69 to 1.96; I^2^ = 0%, *p =* 0.60) and cardiovascular death (0.89, 0.64 to 1.26; I^2^ = 0%, *p =* 0.81) (Fig. 5).

### All-cause death

Eight studies reported 122 deaths in 873 patients with ACEI/ARB treatment (14.0%) and 143 deaths of the 873 patients with placebo or active agents therapy (16.4%) [8–11, 14, 18–20]. Overall, ACEI/ARB therapy did not reduce all-cause mortality of dialysis patients (0.94, 0.75–1.17) (Fig. 5). Subgroup analyses showed that the association between ACEI/ARB therapy and risk of all-cause mortality was not modified by different control group, RASI category, dialysis mode, follow-up year, sample size and patient age (all *p* for heterogeneity >0.05, Additional file 1: Figure S1).

### Decline of residual renal function

**Fig. 6:**
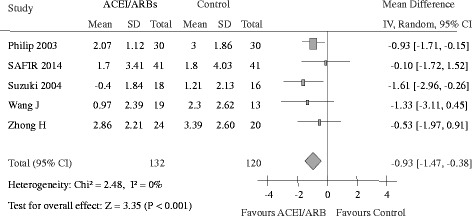
Change of GFR in ACEI/ARB group versus placebo or other active agents group

Data regarding the effects of ACEI/ARB on renal function were available from 5 trials [11, 14–17], including 1 trial (*n* = 82) conducted in hemodialysis patients and 4 in peritoneal dialysis patients (*n* = 170). The average residual GFR declined by 1.44 ml/min per 1.73 m^2^ in the ACEI/ARB group versus 2.37 ml/min per 1.73 m^2^ in the placebo or active control group. The average decline in residual GFR was 0.93 ml/min per 1.73 m^2^ (95% CI, 0.38 to 1.47 ml/min per 1.73 m^2^) less in patients receiving ACEI/ARB than in placebo or active control group patients (*p* < 0.001) with no evidence of heterogeneity (I^2^ = 0%, *p =* 0.65) (Fig. 6).

Three studies including 158 participants reported the changes in urine volume between ACEI/ARB and placebo or active control therapy [11, 17, 18], and found ACEI/ARB treatment was a borderline significant factor in delaying the decline in urine volume: MD 167 ml, 95% CI 21 ml to 357 ml; *p =* 0.08) (Additional file 2: Figure S2).

### Adverse events

Data on adverse events potentially associated with treatment were collected from these studies but were inconsistently reported (Table 3). Overall, ten trials reported at least 1 adverse event. Compared with control, ACE-I/ARBs therapy did not clearly increase the risk of hyperkalemia (1.29, 0.76 to 2.17), hypotension (1.03, 0.73 to 1.45) or cough (2.63, 0.00 to 39,507).

**Table 3:** Adverse events reported in the included RCTs

Adverse Events	Studies Reporting (n)	ACEI/ARB Group (n/n)	Control Group (n/n)	RR (95% CI)	*P* Value
Hyperkalemia	5	33/604	24/605	1.29 (0.76,2.17)	0.34
Hypotension	5	54/604	54/605	1.03 (0.73,1.45)	0.87
Cough	2	3/75	0/77	2.63 (0.00,39,507.62)	0.84

### Risk of bias

Formal statistical testing showed no evidence of publication bias for major cardiovascular events (Begg’s test *p =* 0.87), which was displayed in Additional file 1: Figure S3.

## Discussion

The management of ACEI or ARB in dialysis patients has been an area of intense debate over recent years. In this large quantitative systematic review comprising of 11 trials and 1856 individuals, we demonstrated RAS-Is’ renoprotective effect in patients undergoing dialysis, especially in peritoneal dialysis patients. Subgroup analysis showed ARB treatment exhibited an effect of cardiovascular protection and reduced the risk of heart failure in this population, which appeared to be independent of BP control. No significant difference was observed on the risk of adverse events. Our study provides evidence supporting the protective effect of ACEI or ARB in dialysis patients, especially ARB therapy.

Recent studies have indicated that ACEI or ARB may reduce the rate of CVEs in patients with dialysis, but evidence provided by some studies were underpowered and yielded inconsistent results [8–10]. A large RCT by Suzuki suggested that patients undergoing long-term hemodialysis with ARB have fewer CVEs [10]. In contrast to these beneficial effects of ACEI or ARB on the prevention of CVEs, FOSIDIAL study and OCTOPUS study showed the use of ACEI/ARB did not reduce the incidence of CVEs [9, 19]. In this meta-analysis, no association between ACEI or ARB treatment and fewer CVEs or lower mortality was found. The reason for the decreased relative risk reduction in dialysis patients compared to those with varying degrees of impaired kidney function but not yet dialysis dependent may reflect differences in the distribution of CVEs [21, 22]. Some cardiovascular risk factors in patients on dialysis include disorders of calcium-phosphate and parathyroid hormone, fluid volume overload, anemia, hyperkalemia, increased oxidative stress, and chronic inflammation [23–27]. Many dialysis patients have more than one of these risk factors, leading to an even higher risk of adverse outcomes. These confounding factors could modify the beneficial effect of RAS blockade. These may explain the observations made regarding the negative effect of the ACEI and ARB on cardiovascular disease which was the major determinant of mortality in patients with dialysis.

Subgroup analysis did show that ARB clearly reduced the risk of CVEs including heart failure, suggesting ARB use may still confer benefits to these individuals. Effectiveness of ACEi and ARB in reducing heart failure was only assessed in 4 studies, two of which were negative, thus whether ARB is superior to ACEI in reducing cardiovascular event rates couldn’t be conclusively determined. So far only one head to head study, comparing the effect of ARB and ACEI, did not find ARB to be preferred in dialysis patients at high risk of CVEs [28], however the sample size was relative small. Also, the large study of Fosinopril in Dialysis (FOSIDIAL) evaluated the effect of ACEI on CVEs in our analysis included nearly 400 patients on hemodialysis with relative higher prevalence of left ventricular hypertrophy at baseline in the ACEI group compared with the control group [9]. There was not a significant reduction of CVEs risk by fosinopril detected in the FOSIDIAL study. Therefore, studies with large samples are strongly recommended to confirm the effect of ACEI or ARB on cardiovascular events.

This large and comprehensive meta-analysis in people undergoing dialysis has confirmed the residual renal function protective effects of RAS-Is, especially in patients with peritoneal dialysis. Evidence from Lavoie et al. shows that ARB plays an important role in the amelioration of the development of fibrosis and increasement of peritoneal transport in PD patients, which is in line with reports from some individual studies [29]. Of note, these results are mainly driven by the studies with PD patients, only one study conducted in HD patients [11]. Differences in hydration potentially have impacted the RRF in HD patients. PD and HD may have different effects in terms of fluid volume changes, cardiovascular stability, hydration, and inflammation, which potentially could modify the renoprotective effects of RAAS blockade.

Safety is an important concern with the use of ACEI/ARB in dialysis patients. Previous studies in patients on dialysis showed RAAS-blocking agents therapy was associated with higher risk of developing hyperkalemia and experiencing symptomatic hypotension [30, 31]. Importantly, in the present meta-analysis, we found the incidence of hyperkalemia was not increased in the ACEI/ARB therapy group.In addition the adverse events including hypotension and cough were distributed evenly between ACEI /ARB and control groups. Hence, it seems safe to use ACEI or ARB agents in this patient population.

Our review had a number of strengths. We compared not only cardiovascular outcomes but also residual renal function progression in dialysis patients, including patients on hemodialysis and peritoneal dialysis. Several reviews have evaluated the effect of RASI in dialysis patients. However, these overviews were conducted a few years before without the new trials. A previous systematic review conducted 7 years ago by Davina et al. assessed the cardiovascular outcomes only in 837 hemodialysis patients [32]. Another one conducted by Akbari et al. in patients receiving peritoneal dialysis lacked statistical power to make a definitely determine the effect of RASI with on hard endpoints [33].

Our study does, however, have limitations. Firstly, the majority of studies have been conducted in China or Japan, which has limited the possibility to generalize the results. Secondly, the sample sizes of trials of direct comparison for ACE inhibitors or ARBs were too small to detect a significant difference. The observed different effect between ACEIs and ARBs by indirect comparison should be interpreted with some caution. Thirdly, existence of potential confounding factors could not be excluded. For example, the control group is not homogeneous as it consists by other active agents or placebo, so that different agents might not have the same risk-benefit ratio in patients with dialysis. The limitations of the current study mean that high-quality RCTs with a large sample size are still needed to reliably emphasis the efficacy of ACEIs and ARBs in patients on dialysis.

## Conclusion

This study demonstrates that ACEIs and ARBs therapies decrease the loss of residual renal function, mainly for patients with peritoneal dialysis. Overall, ACE-Is and ARBs do not reduce cardiovascular events in dialysis patients, however, treatment with ARB seems to reduce cardiovascular events including heart failure. ACE-Is and ARBs do not induce an extra risk of side effects. The clinical significance of the results requires confirmation with further studies.

## Additional files

Additional file 1: Figure S1. Subgroup analysis for the relationship between all-cause mortality and the use of ACEI/ARB. (PPTX 73 kb)

Additional file 2: Figure S2. Change of urine volume in ACEI/ARB group versus placebo or other active agents group. (PPTX 70 kb)

Additional file 3: Figure S3. Funnel plots with pseudo 95% confidence limits for CVEs among the included trials. (PPTX 48 kb)

Additional file 4: Search strategy. (DOCX 18 kb)

## Abbreviations

ACEIs: Angiotensin-converting enzyme inhibitors
ARBs: Angiotensin receptor blockers
CI: 95% confidence intervals
CKD: Chronic kidney disease
CVE: Cardiovascular event
ESRD: End-stage renal disease
GFR: Glomerular filtration rate
HD: Hemodialysis
HR: Hazard ratio
PD: Peritoneal dialysis
RCTs: Randomised controlled trials
RR: Risk ratio
RRF: Residual renal Function
