# Supplementary material for: Effects of angiotensin-converting enzyme inhibitors and angiotensin receptor blockers on cardiovascular events and residual renal function in dialysis patients: a meta-analysis of randomised controlled trials
Source: BMC Nephrol. 2017 Jun 30;18:206. doi: 10.1186/s12882-017-0605-7 (PMC5493067; doi:10.1186/s12882-017-0605-7)
Supplement: Supplementary file 1 — Subgroup analysis for the relationship between all-cause mortality and the use of ACEI/ARB. (PPTX 73 kb) [file 12882_2017_605_MOESM1_ESM.pptx]

## Slide 1
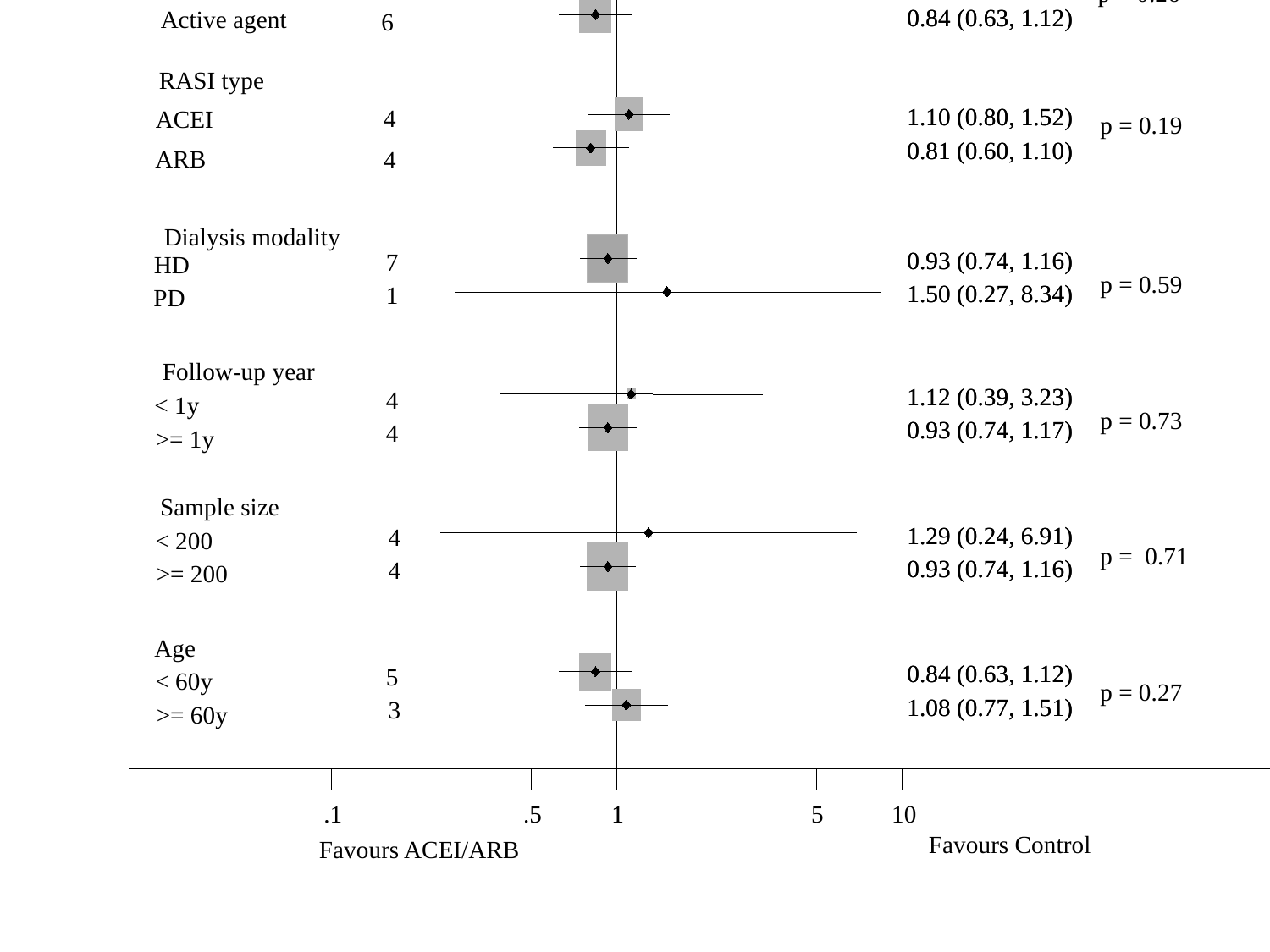

p value for
heterogeneity
No. of trial
Study
Relative ratio (95% CI)
Control group
2
Placebo
1.08 (0.77, 1.52)
1.08 (0.77, 1.52)
p = 0.26
6
0.84 (0.63, 1.12)
0.84 (0.63, 1.12)
Active agent
RASI type
4
1.10 (0.80, 1.52)
1.10 (0.80, 1.52)
ACEI
p = 0.19
0.81 (0.60, 1.10)
0.81 (0.60, 1.10)
4
ARB
Dialysis modality
7
0.93 (0.74, 1.16)
0.93 (0.74, 1.16)
HD
p = 0.59
1
1.50 (0.27, 8.34)
1.50 (0.27, 8.34)
PD
Follow-up year
4
1.12 (0.39, 3.23)
1.12 (0.39, 3.23)
< 1y
p = 0.73
4
0.93 (0.74, 1.17)
0.93 (0.74, 1.17)
>= 1y
Sample size
4
1.29 (0.24, 6.91)
1.29 (0.24, 6.91)
< 200
p = 0.71
4
0.93 (0.74, 1.16)
0.93 (0.74, 1.16)
>= 200
Age
5
0.84 (0.63, 1.12)
0.84 (0.63, 1.12)
< 60y
p = 0.27
3
1.08 (0.77, 1.51)
1.08 (0.77, 1.51)
>= 60y
.1
.5
1
1
5
10
Favours Control
Favours ACEI/ARB
