# Supplementary material for: Effects of angiotensin-converting enzyme inhibitors and angiotensin receptor blockers on cardiovascular events and residual renal function in dialysis patients: a meta-analysis of randomised controlled trials
Source: BMC Nephrol. 2017 Jun 30;18:206. doi: 10.1186/s12882-017-0605-7 (PMC5493067; doi:10.1186/s12882-017-0605-7)
Supplement: Supplementary file 2 — Change of urine volume in ACEI/ARB group versus placebo or other active agents group. (PPTX 70 kb) [file 12882_2017_605_MOESM2_ESM.pptx]

## Slide 1
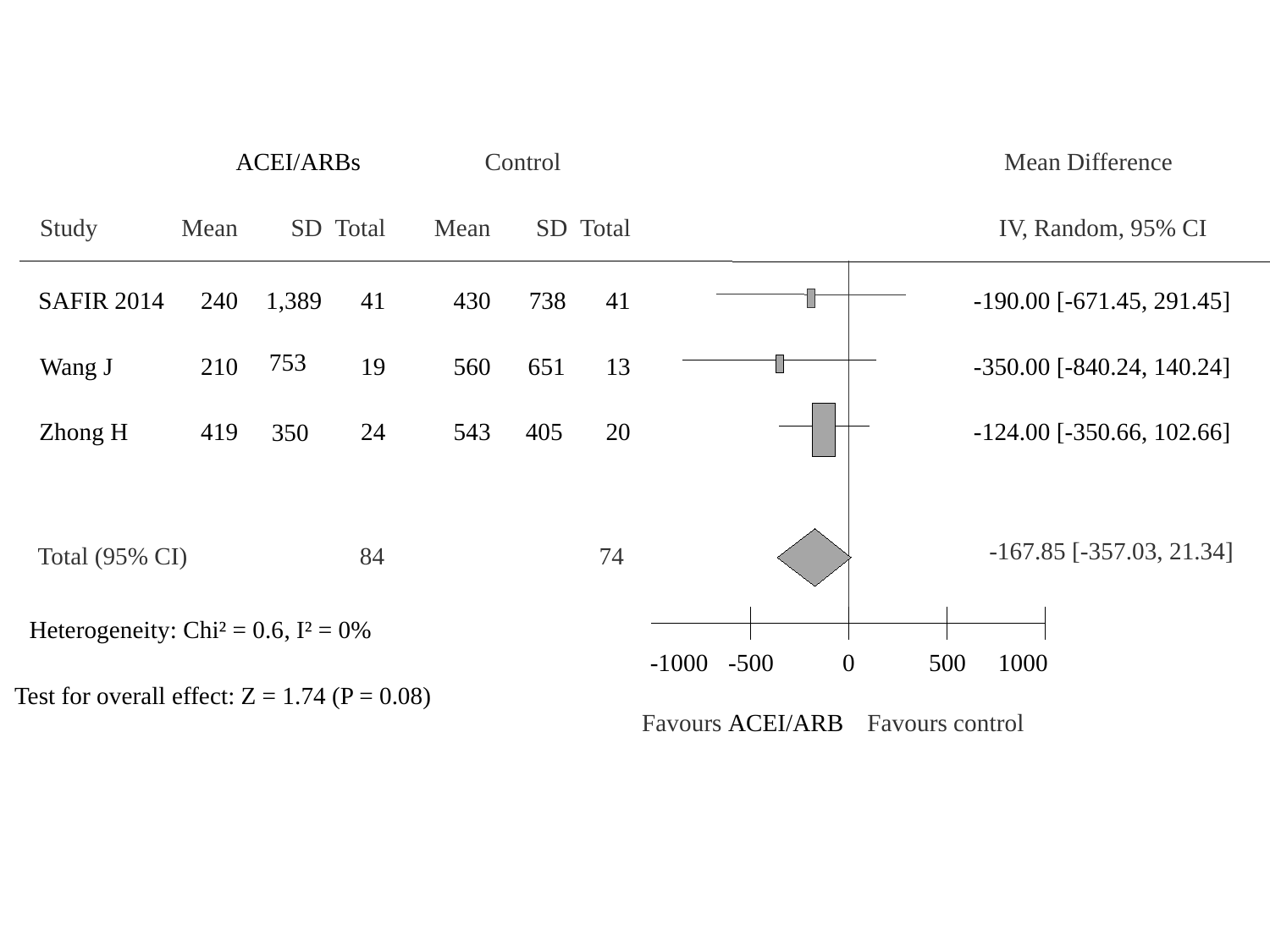

ACEI/ARBs
Control
Mean Difference
Study
Mean
SD
Total
Mean
SD
Total
IV, Random, 95% CI
SAFIR 2014
240
1,389
41
430
738
41
-190.00 [-671.45, 291.45]
753
Wang J
210
19
560
651
13
-350.00 [-840.24, 140.24]
Zhong H
419
24
543
405
20
-124.00 [-350.66, 102.66]
350
-167.85 [-357.03, 21.34]
Total (95% CI)
84
74
Heterogeneity: Chi² = 0.6, I² = 0%
-1000
-500
0
500
1000
Test for overall effect: Z = 1.74 (P = 0.08)
Favours ACEI/ARB
Favours control
