# Supplementary figures and images for: Effects of angiotensin-converting enzyme inhibitors and angiotensin receptor blockers on cardiovascular events and residual renal function in dialysis patients: a meta-analysis of randomised controlled trials
Source: BMC Nephrol. 2017 Jun 30;18:206. doi: 10.1186/s12882-017-0605-7 (PMC5493067; doi:10.1186/s12882-017-0605-7)

## Slide 1
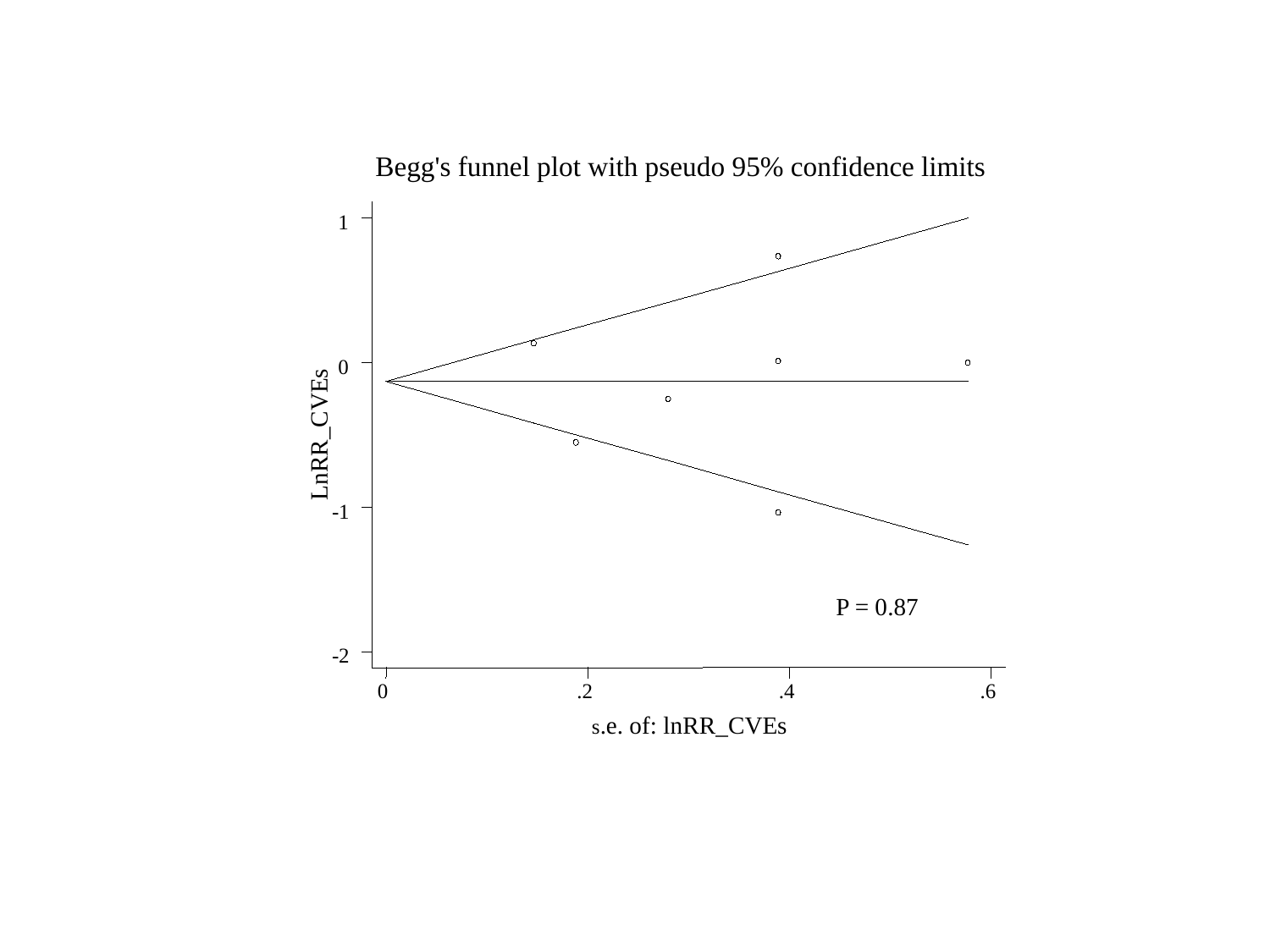

Begg's funnel plot with pseudo 95% confidence limits
1
0
LnRR_CVEs
-1
P = 0.87
-2
0
.2
.4
.6
s.e. of: lnRR_CVEs

Supplement: Supplementary file 3 — Funnel plots with pseudo 95% confidence limits for CVEs among the included trials. (PPTX 48 kb) [file 12882_2017_605_MOESM3_ESM.pptx]
