# Supplementary material for: Effects of angiotensin-converting enzyme inhibitors and angiotensin receptor blockers on cardiovascular events and residual renal function in dialysis patients: a meta-analysis of randomised controlled trials
Source: BMC Nephrol. 2017 Jun 30;18:206. doi: 10.1186/s12882-017-0605-7 (PMC5493067; doi:10.1186/s12882-017-0605-7)
Supplement: Supplementary file 4 — Search strategy. (DOCX 18 kb) [file 12882_2017_605_MOESM4_ESM.docx]

The search strategy

MEDLINE (OVID)

1. exp Angiotensin-Converting Enzyme Inhibitors/

2. angiotensin converting enzyme inhibit$.tw.

3. (ace adj2 inhibit$).tw.

4. (ACE or ACE1 or ACEI or ACE-I or ACEs).

5.alacepril.tw.

6.benazepril.tw.

7.benazeprila.tw.

8. captopril.tw.

9. enalapril.tw.

10. cilazapril.tw.

11. enalaprilat.tw.

12. fosinopril.tw.

13. lisinopril.tw.

14. perindopril.tw.

15. ramipril.tw.

16. benazeprilat.tw.

17. ceranapril.tw.

18. cilazaprilat.tw.

19. delapril.tw.

20 .fosinoprilic acid.tw.

21. imidapril.tw.

22. libenzapril.tw.

23. quinaprilat.tw.

24. ramiprilat.tw.

25. rentiapril.tw.

26. saralasin.tw.

27. spirapril.tw.

28.temocapril hydrochloride

29. teprotide.tw.

30. zofenopril.tw.

31. trandolapril.tw.

32.quinapril.tw.

33.moexipril.tw.

34.ceronapril.tw.

35. deacetylalacepril.tw.

36.fosinopril$.

37.losartan.tw.

38.azilsartan.tw.

39.elisartan.tw

40.embusartan.tw.

41.forasartan.tw.

42.saprisartan.tw.

43.tasosartan.tw.

44.vasvalsartan.tw.

45.zolasartan.tw.

46.miardis.tw.

47.cozaar.tw.

48.atacand.tw.

49.teveten.tw.

50.avapro.tw.

51.micardis.tw.

52.avalide.tw.

53.aprovel.tw.

54.amias.tw.

55.diovan.tw.

56.olmetec.tw.

57. irbesartan.tw.

58. candesartan.tw.

59. eprosartan.tw.

60. valsartan.tw.

61. olmesartan.tw.

62. telmisartan.tw.

63. (ace adj2 inhibitor$).tw.

64. (angiotensin adj2 receptor antagonist$).tw.

65.ARB.tw.

66.Angiotensin 2 Receptor Antagonist.tw.

67.Angiotensin Receptor Antagonist.tw.

68.Angiotensin II Antagonist.tw.

69.AT 2 receptor blocker.tw.

70.AT 2 receptor antagonist.tw.

71. or/1-70

72. Kidney Failure, Chronic/

73. Kidney Failure/

74. Renal Insufficiency, Chronic/ or Kidney Failure, Chronic/

75. Uremia/

76. (CKF or CRF or ESRD or ESKF or ESRF or ESKD).tw.

77. (end-stage renal or end-stage kidney or endstage renal or endstage kidney).tw.

78. renal replacement therapy/ or exp renal dialysis/ or exp hemofiltration/

79. (predialysis or dialysis).tw.

80. (haemodialysis or haemodialysis).tw.

81. (hemofiltration or haemofiltration).tw.

82. (hemodiafiltration or haemodiafiltration).tw.

83. (CAPD or CCPD or APD or PD).tw.

84. or/72-83

85. Randomized controlled trial.pt.

86.Controlled clinical trial.pt

87. Randomized.tw.

88. Placebo.tw.

89.Drug therapy/

90.Randomly.tw.

91.Trial.tw.

92.Groups.tw.

93.(random$ adj5 trial$).tw.

94. (random$ adj5 allocation$).tw.

95.(Blind$ adj5 method$).tw.

96.or/85-95

97. 71 and 84 and 96

EMBASE

1. exp Angiotensin-Converting Enzyme Inhibitors

2. angiotensin adj2 receptor antagonist$

3. Angiotensin 2 Receptor Antagonist

4. Angiotensin Receptor Antagonist

5 . Angiotensin II Antagonist

6. AT 2 receptor blocker

7. AT 2 receptor antagonist

8. fosinopril$

9. losartan

10. azilsartan

11. elisartan

12. embusartan

13. forasartan

14. saprisartan

15. tasosartan

16. vasvalsartan

17. zolasartan

18. miardis

19. cozaar

20. atacand

21. teveten

22. avapro

23. micardis

24. avalide

25. aprovel

26. amias

27. diovan

28. olmetec

29. irbesartan

30. candesartan

31. eprosartan

32. valsartan

33. olmesartan

34. telmisartan

35. ace adj2 inhibitor$

36. angiotensin converting enzyme inhibit$

37. ace adj2 inhibit$

38. ACE or ACE1 or ACEI or ACE-I or ACE

39.alacepril

40.benazepril

41.benazeprila

42. captopril

43. enalapril

44. cilazapril

45. enalaprilat

46. fosinopril

47. lisinopril

48. perindopril

49. ramipril

50. benazeprilat

51. ceranapril

52. cilazaprilat

53. delapril

54. fosinoprilic acid

55. imidapril

56. libenzapril

57. quinaprilat

58. ramiprilat

59. rentiapril

60. saralasin

61. spirapril

62.temocapril hydrochloride

63. teprotide

64. zofenopril

65. trandolapril

66.quinapril

67.moexipril

68.ceronapril

69. deacetylalacepril

70 .or/1-69

71. Kidney Failure, Chronic

72. Kidney Failure

73. Uremia

74. CKF or CRF or ESRD or ESKF or ESRF or ESKD

75. end-stage renal or end-stage kidney or endstage renal or endstage kidney

76. predialysis or dialysis

77. haemodialysis or haemodialysis

78. hemodiafiltration or haemodiafiltration

79. CAPD or CCPD or APD or PD

80. or/72-87

81. Randomized controlled trial

82. Controlled clinical trial

83. blind and method

84. randomized and controlled and trial

85. random and allocation

86. or/89-93

87. 70 and 80 and 86

COCHRANE CONTROLLED TRIALS

1. exp Angiotensin-Converting Enzyme Inhibitors/

2. angiotensin converting enzyme inhibit$.tw.

3. (ace adj2 inhibit$).tw.

4. (ACE or ACE1 or ACEI or ACE-I or ACEs).tw.

5. alacepril.tw.

6. benazepril.tw.

7. benazeprila.tw.

8. captopril.tw.

9. enalapril.tw.

10. cilazapril.tw.

11. enalaprilat.tw.

12. fosinopril.tw.

13. lisinopril.tw.

14. perindopril.tw.

15. ramipril.tw.

16. benazeprilat.tw.

17. ceranapril.tw.

18. cilazaprilat.tw.

19. delapril.tw.

20 .fosinoprilic acid.tw.

21. imidapril.tw.

22. libenzapril.tw.

23. quinaprilat.tw.

24. ramiprilat.tw.

25. rentiapril.tw.

26. saralasin.tw.

27. spirapril.tw.

28.temocapril hydrochloride

29. teprotide.tw.

30. zofenopril.tw.

31. trandolapril.tw

32. quinapril.tw.

33. moexipril.tw.

34. ceronapril

35. deacetylalacepril.tw.

36. fosinopril$.

37. losartan.tw.

38. azilsartan.tw.

39. elisartan.tw

40. embusartan.tw.

41. forasartan.tw.

42. saprisartan.tw.

43. tasosartan.tw.

44. vasvalsartan.tw.

45. zolasartan.tw.

46. miardis.tw.

47. cozaar.tw.

48. atacand.tw.

49. teveten.tw.

50. avapro.tw.

51. micardis.tw.

52. avalide.tw.

53. aprovel.tw.

54. amias.tw.

55. diovan.tw.

56. olmetec.tw.

57. irbesartan.tw.

58. candesartan.tw.

59. eprosartan.tw.

60. valsartan.tw.

61. olmesartan.tw.

62. telmisartan.tw.

63. (ace adj2 inhibitor$).tw.

64. (angiotensin adj2 receptor antagonist$).tw.

65. ARB.tw.

66. Angiotensin 2 Receptor Antagonist.tw.

67. Angiotensin Receptor Antagonist.tw.

68. Angiotensin II Antagonist.tw.

69. AT 2 receptor blocker.tw.

70.AT 2 receptor antagonist.tw.

71. or/1-70

72. Kidney Failure, Chronic/

73. Kidney Failure/

74. Renal Insufficiency, Chronic/ or Kidney Failure, Chronic/

75. Uremia/

76. (CKF or CRF or ESRD or ESKF or ESRF or ESKD).tw.

77. (end-stage renal or end-stage kidney or endstage renal or endstage kidney).tw.

78. renal replacement therapy/ or exp renal dialysis/ or exp hemofiltration/

79. (predialysis or dialysis).tw.

80. (haemodialysis or haemodialysis).tw.

81. (hemofiltration or haemofiltration).tw.

82. (hemodiafiltration or haemodiafiltration).tw.

83. (CAPD or CCPD or APD or PD).tw.

84. or/72-83

85. Randomized controlled trial.pt.

86. Controlled clinical trial.pt

87. Randomized.tw.

88. Placebo.tw.

89. Drug therapy/

90. Randomly.tw.

91.Trial.tw.

92. Groups.tw.

93. (random$ adj5 trial$).tw.

94. (random$ adj5 allocation$).tw.

95. (Blind$ adj5 method$).tw.

96. or/85-95

97. 71 and 84 and 96
